# A Novel Acute Retroviral Syndrome Severity Score Predicts the Key Surrogate Markers for HIV-1 Disease Progression

**DOI:** 10.1371/journal.pone.0114111

**Published:** 2014-12-09

**Authors:** Dominique L. Braun, Roger Kouyos, Corinna Oberle, Christina Grube, Beda Joos, Jacques Fellay, Paul J. McLaren, Herbert Kuster, Huldrych F. Günthard

**Affiliations:** 1 Division of Infectious Diseases and Hospital Epidemiology, University Hospital Zurich, University of Zurich, Zurich, Switzerland; 2 School of Life Sciences, École Polytechnique Fédérale de Lausanne, Lausanne, Switzerland; Institute of Microbiology, University Hospital Center and University of Lausanne, Lausanne, Switzerland; Centro Nacional de Microbiología - Instituto de Salud Carlos III, Spain

## Abstract

***Objective:*** Best long-term practice in primary HIV-1 infection (PHI) remains unknown for the individual. A risk-based scoring system associated with surrogate markers of HIV-1 disease progression could be helpful to stratify patients with PHI at highest risk for HIV-1 disease progression.

***Methods:*** We prospectively enrolled 290 individuals with well-documented PHI in the Zurich Primary HIV-1 Infection Study, an open-label, non-randomized, observational, single-center study. Patients could choose to undergo early antiretroviral treatment (eART) and stop it after one year of undetectable viremia, to go on with treatment indefinitely, or to defer treatment. For each patient we calculated an a priori defined “Acute Retroviral Syndrome Severity Score” (ARSSS), consisting of clinical and basic laboratory variables, ranging from zero to ten points. We used linear regression models to assess the association between ARSSS and log baseline viral load (VL), baseline CD4^+^ cell count, and log viral setpoint (sVL) (i.e. VL measured ≥90 days after infection or treatment interruption).

**Results:**

Mean ARSSS was 2.89. CD4^+^ cell count at baseline was negatively correlated with ARSSS (p = 0.03, n = 289), whereas HIV-RNA levels at baseline showed a strong positive correlation with ARSSS (p<0.001, n = 290). In the regression models, a 1-point increase in the score corresponded to a 0.10 log increase in baseline VL and a CD4^+^cell count decline of 12/µl, respectively. In patients with PHI and not undergoing eART, higher ARSSS were significantly associated with higher sVL (p = 0.029, n = 64). In contrast, in patients undergoing eART with subsequent structured treatment interruption, no correlation was found between sVL and ARSSS (p = 0.28, n = 40).

**Conclusion:**

The ARSSS is a simple clinical score that correlates with the best-validated surrogate markers of HIV-1 disease progression. In regions where ART is not universally available and eART is not standard this score may help identifying patients who will profit the most from early antiretroviral therapy.

## Introduction

Since 2010, HIV treatment guidelines recommend early antiretroviral therapy (eART) in case of symptomatic primary HIV-infection (PHI) [1], fostered by increasing evidence that immediate treatment is beneficial for patients with PHI [2]–[6]. On an individual level, however, best long-term practice in PHI remains uncertain and the value of eART is still debated due to controversial results reported in literature [2]–[4], [6]–[9]. Several studies report a relationship between the severity of PHI and disease progression and death [10]–[12]. Based on these data it is convincing that patients with severe manifestation of PHI probably benefit the most from prompt initiation of ART. On the other hand, there exist no clear definition of severe PHI and withholding ART to study the natural history of the HIV-infection has been unethical for almost two decades. We therefore developed the Acute Retroviral Syndrome Severity Score (ARSSS), which includes clinical symptoms and a few general laboratory parameters that are regularly obtained in a routine primary care setting for patients presenting with PHI. We hypothesized that the intensity of the clinical presentation of PHI expressed by our newly developed ARSSS correlates with the best validated surrogate markers associated with HIV-1 disease progression [11], [13], [14]. Our aim was to create an easy obtainable risk score which could help clinicians to identify patients who might profit most of eART, in particularly in regions where universal ART is not available. We evaluated our ARSSS in 290 individuals with a well-documented PHI within the frame of the Zurich Primary HIV-infection study.

## Study Design, Patients and Methods

### Study design and patient selection

Between January 2002 and September 2012 we prospectively enrolled 290 individuals with a documented PHI in the longitudinal Zurich Primary HIV Infection Study (ZPHIS), which is an open-label, non-randomized, observational, single-center study (http://clinicaltrials.gov, ID 5 NCT00537966) [5], [6], [15]. All patients ≥18 years who fulfilled the inclusion criteria of a documented acute or recent HIV infection (definition see below) and who gave their informed consent were included in the study. Patients could choose to undergo eART and stop it after one year of undetectable viremia (<50 copies HIV-1 RNA/ml plasma), to go on with treatment indefinitely, or to defer treatment. During the first visit, a detailed history of symptoms and signs of the acute retroviral syndrome (ARS) was obtained, as well as a physical examination and standard laboratory parameters (including full blood count and chemistry in addition to specific HIV-1 laboratory parameters such as HIV-1 viral load, CD4^+^ cell count, HIV-1 Immunoblot, p24 antigen and genotypic resistance testing). Patients were actively screened for acute hepatitis B and C, syphilis, gonorrhea, chlamydia trachomatis and herpes simplex. If the patient was referred from an external physician, data from the first external visit were recorded.

### Ethics Statement

The ethic committee of the University Hospital Zurich approved the study protocol and a written informed consent was obtained from all patients.

### Definition of acute and recent primary HIV-infection and of estimated date of infection

Acute/recent PHI was confirmed in all patients as previously published [6], [15], [16]: acute HIV-infection was defined as ARS and negative or indeterminate Westernblot in the presence of a positive p24-antigen and/or detectable HIV-1 RNA; or as a documented seroconversion with a 4^th^ generation HIV screening test with or without symptoms during the past 90 days. Recent infection was defined as possible ARS, positive Westernblot and detectable HIV-RNA and a negative HIV-gp120 avidity or detuned assay; or as a documented acute HIV-1 infection with referral to our center more than 90 days after presumed date of infection. For each patient an estimated date of infection (EDI) was determined as previously described [5], [6], [15] by taking into account the pattern of different assay reactivity's (first positive and last negative HIV test, negative, indeterminate and positive WB, positive p24 antigen, and avidity assay), patient's reports of unambiguous risk contacts, and timing of onset of ARS symptoms.

### Antiretroviral treatment and follow-up

Since the beginning of the study in 2002, eART was offered to all patients in a research setting, even though general treatment guidelines did not yet recommend eART for PHI patients [17]. Over time these treatment recommendations have changed towards treating HIV-infected individuals regardless of their CD4^+^ cell count, including patients with PHI [1], [18]. The initial treatment consisted of a ritonavir-boosted protease inhibitor (PI) combined with two nucleoside reverse transcriptase inhibitors (nRTI) (e.g. lopinavir/ritonavir or darunavir/ritonavir in combination with zidovudine/lamivudine or emtricitabine/tenofovir) reflecting the introduction of these drugs into clinical practice [1], [17], [18]. Treatment was continued for at least 12 months when plasma HIV- RNA was <50 copies/ml. After one year of suppressed viremia, patients could choose to stop ART. Of note, structured treatment interruption was no longer recommended since 2010 due to increasing evidence that effect of eART after treatment stop is only transient and vanishes over the time [19] and due to a change in treatment recommendations towards universal treatment [1]. In the case of structured treatment interruption, reintroduction of cART was recommended to the patients if CD4^+^ cell count dropped to below 350 copies/ml [1], [20]. After cessation of cART, the VL and CD4^+^ cell counts were collected every 2-4 weeks during the first three months and then on every regular visit four times annually.

### Acute retroviral syndrome

Based on an extensive literature search we defined 17 symptoms and signs which we considered ARS symptoms and signs (Table 1) [21]–[31]. An ARS was stated in the presence of fever plus at least one of the 17 symptoms/signs or, in absence of fever, ≥2 symptoms/signs.

**Table 1:** Symptoms of acute retroviral syndrome reported in eleven retrospective studies or review articles.

Symptoms/signs of ARS	Median %	Range %
Fever	78	23–100
Skin rash	38	4–75
Pharyngitis	48	2–95
Lymphadenopathy	44	7–75
Myalgia	46	14–92
Headache	44	18–58
Diarrhea	32	14–48
Arthralgias	27	5–72
Cough	25	4–45
Nausea	32	6–67
Malaise/fatigue	64	12–92
Vomiting	32	3–67
Weight loss	21	2–46
Genital ulcer	3	3–10
Oral ulcers	17	9–30
Aseptic Meningitis	12	0–24
Night sweat	14	9–48

### Acute Retroviral Syndrome Severity Score

We intended to develop an ARSSS which could be calculated easily within a short time without including time-consuming, expensive, or difficult to perform laboratory analysis (Table 2). The ARSSS was defined a priori. We intentionally did not perform separate analyses of individual variables. The six variables and their weight were chosen based on epidemiological and clinical evidence and our own profound experience with PHI. For each patient we calculated an ARSSS ranging from zero to 10 points. The rational for using the six scoring variables was based on following considerations and evidence: (i) Severe neurological symptoms, inpatient treatment, fever and age >50 years: it has been shown that patients with prolonged or symptomatic manifestation of primary HIV infection and older HIV patients have an increased risk of HIV-1 disease progression [10]–[12], [32], [33]. Of note, a recent publication showed that encephalitis and meningitis are a surrogate for severe PHI and predict faster disease progression [11]. (ii) Low platelet count and elevated aminotransferases: these laboratory markers have been demonstrated to predict faster disease progression in patients with primary and chronic HIV-infection [11], [34]–[36].

**Table 2:** Acute Retroviral Syndrome Severity Score (ARSSS).

Parameters	Related scoring point(s)
Severe neurological symptoms^a^	3
Inpatient treatment	3
Age ≥50 years	1
Fever (self-reported or documented ≥38° degrees Celsius)	1
Elevated liver enzymes (ASAT and/or ALAT ≥30 U/l)	1
Thrombocytopenia (platelet count <150 G/l)	1
**Maximum value**	**10**

a e.g. encephalitis, aseptic meningitis, paresis, facial nerve paresis

### Baseline CD4^+^ cell count, baseline viral load and viral setpoint

Baseline VL and baseline CD4^+^ cell count were defined as the first values available. The viral setpoint (sVL) was defined as the first HIV-RNA measurement ≥90 days after the EDI in treatment-naïve patients and ≥90 days after controlled treatment interruption in patients with eART [6]. The cutoff point of ≥90 days after the EDI was chosen based on the observation that the initially very high levels of viral HIV-1 RNA and DNA, which are a hallmark of acute HIV-1 infection, appear to level off after approximately 3 months post-infection [6].

### Genome-wide association analysis

Genomic DNA samples were genotyped using the HumanOmniExpress chip (Illumina), which features >700,000 single nucleotide polymorphisms (SNPs). SNPs were filtered based on missingness (dropped if called in <98% of participants), minor allele frequency (dropped if <1%) and severe deviation from Hardy–Weinberg equilibrium (dropped if p<10^6^). High-resolution HLA class I typing was imputed from the SNP data [37]. We used linear regression to test for association between genetic variants and ARSSS. To avoid spurious associations resulting from population stratification, we used a modified Eigenstrat method, which derives the principal components of the correlations among SNPs [38]: population outliers were discarded, and the coordinates of the significant principal component axes were included in the association tests to correct for residual stratification. Bonferroni's correction was applied for multiple testing.

### Statistical analysis

We used linear regression models to assess the impact of ARSSS on log10 VL. Since the baseline VL was strongly associated with the time-point of measurement (i.e. days after EDI), we corrected for the timepoint of VL measurement in a multivariable regression model. This model included both the ARSSS and the time between the viral load measurement and the EDI as explanatory variables. Specifically, we included time both as a linear and as quadratic term in order to capture the non-monotonic changes of virus load over time. The impact of ARSSS on treatment initiation was assessed using Cox-proportional hazard models (with event  =  “treatment initiation”) and logistic regression (with outcome  = “patient started treatment within a given time-window”).

## Results

### Patient characteristics

We analysed 290 individuals with a documented PHI, including 271 males. Self-reported transmission modes included men who have sex with men (MSM) (78%), heterosexual (20%), intravenous drug abuse (IVDA) (1%) and others (1%). The most prevalent HIV-subtype was B (76%), followed by CRF01_AE (7%), A (4%) and C (2%). 23 patients (8%) showed ≥ one mutation associated with transmitted drug resistance (TDR). Of all individuals, 17% presented with signs of a concomitant STI at presentation. 141 patients (49%) first consulted their primary physician, 69 (24%) the hospital and 42 (14%) an outpatient unit (Table 3).

**Table 3:** Baseline characteristics of 290 patients with primary HIV-1 infection.

	Total patients		Female		Acute infection		Recent infection	
	n	% or range	n	%	n	%	n	%
**Number of patients**	290	100	19	7	242	83	48	17
Male	271	93			228	93	44	96
Female	19	7			16	7	2	4
**Age (years)**	36	18–70	34	19–55	36	18–70	35	19–63
**HIV-1 Subtype B** a	220	76	5	26	179	77	37	80
**Transmission mode**								
MSM	225	78			185	76	40	87
Heterosexual	59	20	19	100	51	22	6	13
IVDU	4	1			5	2		
Others b	2	1			1	1	3	7
**STIs** c	49	17	1	5	36	15	12	26
**Initial presentation**								
General practitioner	141	49	7	37	119	49	22	45
Hospital	69	24	8	42	58	23	11	28
Outpatient unit	42	14	2	11	41	18	1	2
Others^d^	38	12	22	11	24	10	14	25
**TDR**	23	8	0	0	21	9	2	4
**Fiebig stages** e								
I/II	3	1.0			3	1.0		
II–III	48	17	3	16	48	20		
IV–VI	218	75	14	74	174	72	42	92
**Median baseline viral load** log10 RNA	6.6	1.8–8	6.1	3.6–7	6.7	1.8–8	5.3	2.4–6.4
**Median baseline CD4 cell count** cells/µl	429	75–1255	443	133–840	412	75–1240	516	164–1255

Abbreviations: MSM: men who have sex with men; IVDU, intravenous drug users; STIs, sexually transmitted infections; HIV-1, human immunodeficiency virus type 1; TDR, transmitted drug resistance.

a Other subtypes: CRF01_AE, C, A, F1, G, CRF02_AG, CRF14_BG, A1D, CR 12_BF, D

b One case from a needle stick.

c Concomitant STIs: syphilis and/or chlamydia and/or gonorrhoea and/or genital herpes

d Non infectious disease specialist or other institutions (e.g. dermatologist, gynaecologist, blood donation center etc.).

e In 21 patients a Fiebig stage could not be assigned due to missing p24-antigen values.

### Acute primary HIV infection and estimated date of infection

PHI was classified in 242 (83%) individuals as “acute” and in 48 (17%) as “recent” infection. Of the acutely and recently infected patients, 226 of 242 (93%) and 31 of 48 (65%) individuals presented with an ARS, respectively. Of all 290 patients, 16 patients (6%) presented without any symptoms and were diagnosed by routine HIV testing. Seventeen patients (6%) presented with symptoms and signs not considered as ARS. Patients were also classified according to the widely used Fiebig stages (Table 2), which are based on the sequential detectability of a number of direct and indirect HIV-1 diagnostic tests (e.g. plasma HIV-1 RNA, p24antigen, HIV-1-specific antibodies detected by ELISA and by western blot) [39]. The mean time between the EDI and the first positive HIV-test overall was 43 days (range 4 to 180 days).

### Acute Retroviral Syndrome Severity Score

**Figure 1:**
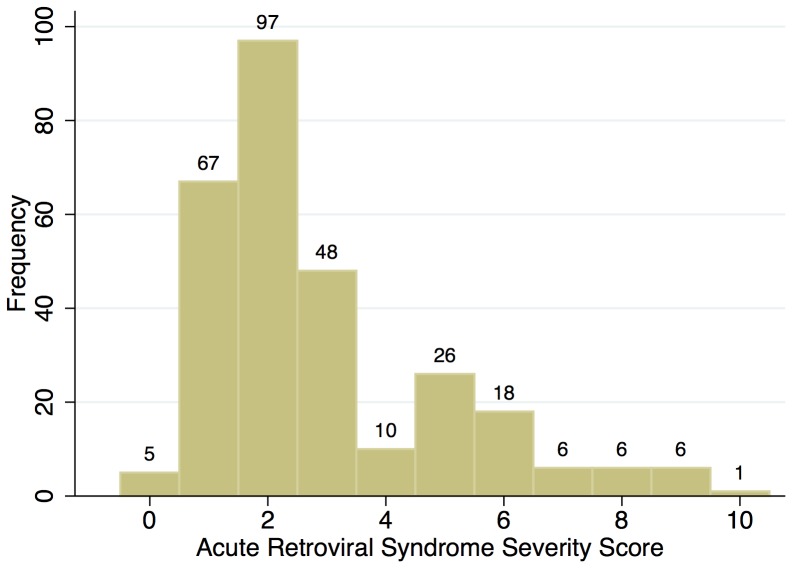
Distribution of scoring points matched to the study subjects. The ARSSS consists of six variables (see Table 1) with an individual count of scoring points for each variable. The mean ARSSS in 290 study subjects was 2.89 points, the majority of them had an ARSSS between 1–3 points.

For the majority (70%) of included individuals, ARSSS-values were between 1 and 3 points, with a mean of 2.89 (range 0 to 10; Fig. 1). 52 patients with PHI (18%) were treated as inpatients. 41 patients (14%) presented with severe neurological symptoms (e.g., encephalitis, aseptic meningitis, facial nerve paresis); of those, 18 (35%) required hospitalisation. Fever was the most common clinical sign of ARS in 240 of 257 patients (93%), followed by malaise in 167 (65%), pharyngitis in 136 (53%), skin rash in 123 (48%), lymphadenopathy in 121 (47%) and diarrhoea in 91 (35%). Elevated liver enzymes and thrombocytopenia were present in 61% and 35%, respectively.

### Association of ARSSS with baseline CD4^+^ cell count, baseline viral load, and viral setpoint

#### Baseline viral load

In univariable analysis, HIV-RNA levels at baseline increased significantly with increasing ARSSS (p<0.001, n = 290). This analysis may be confounded by the time of viral load measurement, because during PHI the HIV-plasma RNA values are highly dynamic and change rapidly [40]. Thus, in the multivariable analysis we corrected for this potential bias by including the EDI. After correction, the association between ARSSS and baseline viral load remained highly significant (p<0.001).

#### Baseline CD4^+^ cell count

**Figure 2:**
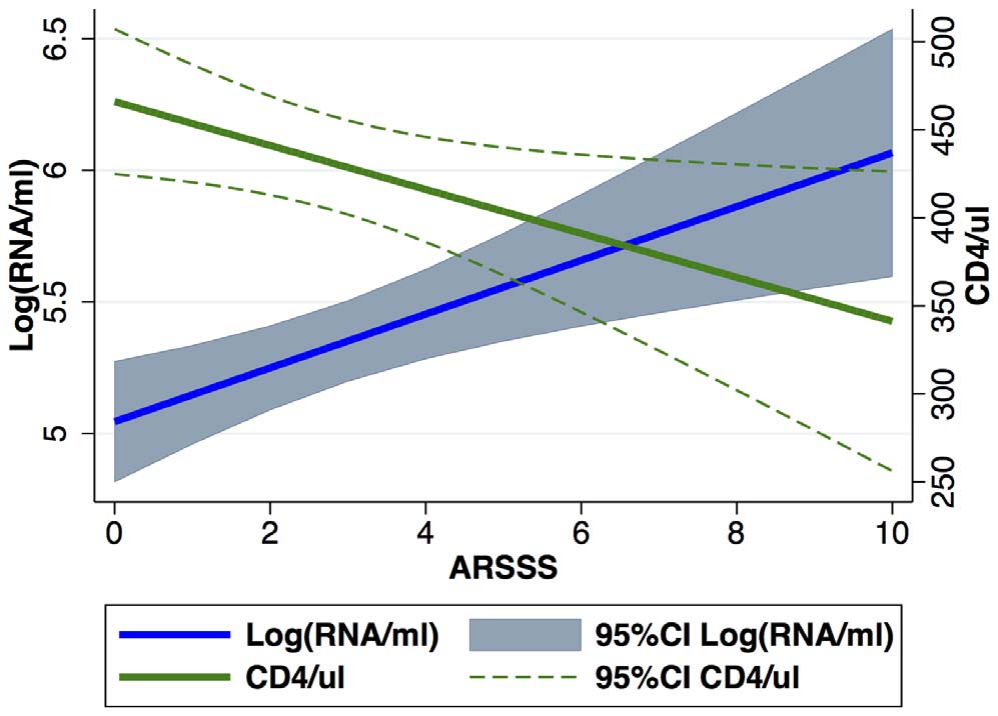
Relationship between the ARSSS and the log HIV-1 RNA (copies/ml plasma) and the CD4^+^ cell count after an estimated duration of infection of 90 days. There is an inverse correlation between CD4^+^ cell count (green line) and the HIV-1 viral load (blue line) and the level of ARSSS. The grey area indicates the 95% confidence interval of the viral load and the green dotted line that of the CD4^+^ cell count, respectively.

In univariable analysis, an increasing ARSSS was inversely correlated with baseline CD4^+^ cell count (p = 0.03, n = 289). The same result was found in multivariable analysis corrected for time (p = 0.03; n = 289). In the above models, there was a significant association between ARSSS and CD4+ cell count and viral load, respectively: In multivariable models, an increase of one scoring point corresponded to a 0.10 log increase in baseline VL and a CD4^+^cell count decline of 12 cells/µl, respectively (Fig. 2).

#### Setpoint viral load in patients without eART

Similar results were found for patients without eART (n = 64). In this subset, individual sVL after 90 days of infection were calculated, showing that higher ARSSS were significantly associated with higher sVL (p = 0.029).

#### Setpoint viral load in patients after controlled treatment interruption of eART

In contrast to untreated patients, no significant correlation between ARSSS and the level of the sVL was found after controlled treatment interruption (p = 0.28, n = 40).

### ARSSS and time to treatment and HIV-1 RNA suppression

To test the hypothesis that individuals with a high ARSSS were treated faster, we investigated whether there was a correlation between an ARSSS above the median value and the time to treatment (i.e. time period between the first positive test and the start of treatment). Indeed, these individuals were treated significantly faster than individuals with an ARSSS below the median value (p = 0.004 in a Cox-proportional hazard model with start of ART as an outcome). The impact of having an ARSS above the median value remained significant when we considered whether patients received treatment within a given time-window (using logistic regression with “receiving treatment within 7 days after positive HIV test” as outcome: p = 0.05, OR [95%CI]  = 1.8 [1.0, 3.3]; within 14 days: p<0.001, OR [95%CI]  = 2.5 [1.5, 4.0]; within 28 days: p = 0.01, OR [95%CI]  = 2.2 [1.2, 4.0]; within 60 days: p = 0.001, OR [95%CI]  = 5.3 [1.8, 16]; or within 84 days: p = 0.002, OR [95%CI]  = 4.8 [1.6, 14.3]). After adjusting for CD4^+^ cell values at baseline, the association between ARSSS and time to treatment persists and the magnitude of the association is only slightly weaker: for 7 days 1.6 [0.9, 3.0]; for 14 days 2.2 [1.3, 3.6]; for 28 days 2.0 [1.1,3.6]; for 60 days 4.7 [1.6,13.9]; and for 84 days 4.2 [1.4, 12.5]. After ZPHI enrollment, 171 of all 290 patients (59%) started an eART within one day. However, patients with an ARSSS above the median score were significantly more likely to start an eART within one day than individuals with an ARSSS below the median value (p = 0.04). Within 7 days, 222 of the 290 (77%) enrolled study subjects had started eART. No correlation was found between the ARSSS and time to virus suppression.

### Genetics and immunogenetics of ARSSS

Genome-wide genotyping was performed for 250 patients. After SNP quality control and exclusion of population outliers, 652,904 SNPs were available for association testing, from 196 patients of European ancestry. After correction for multiple testing, no association between ARSSS and human SNPs or HLA class I types was found. In particular, the SNPs tagging HLA-B*5701 (rs2395029) and HLA-C expression levels (rs9264942), which are the strongest genetic predictors of HIV-1 control [41], did not discriminate between patients with high or low ARSSS.

## Discussion

The most relevant finding of this study is that the newly developed ARSSS, a risk stratification score based on simple and easily obtainable clinical and laboratory parameters, is predictive of the two major, well studied, and clinically relevant surrogate markers associated with HIV-1 disease progression. In particular, a significant correlation was found between the ARSSS and the baseline CD4^+^ cell count, the baseline viral load, and the setpoint viral load in untreated patients with a primary HIV-1 infection.

Although a vast number of potential surrogate markers for HIV-1 disease progression have been studied [42]–[44] - almost exclusively in chronically infected patients - to date the two most important parameters in clinical practice remain CD4^+^ cell count and viral load [13]. These two parameters have been validated in very large datasets in regard to AIDS defining, non-AIDS defining clinical endpoints and in regard to mortality [13], [45], [46]. Contrary to chronic HIV-infection, in PHI the value of the surrogate markers CD4^+^ cell count and viral load is less studied and validated due to inherent difficulties in diagnosing and recruiting patients with PHI in large numbers and to the considerable variation of immunological and virological parameters during PHI [40]. However, increasing evidence shows that both a low initial CD4 cell count and a high HIV-RNA level are predictive for rapid progression of untreated primary HIV infection [2], [4], [47]. A recent report from Lodi et al. showed that a CD4^+^ cell count <350 cell/µl within six months of seroconversion was associated with a significant increased risk for AIDS and death in patients with PHI in the preART era [11]. Contrary to our work, the baseline viral load was not included as predictor in this analysis. Apart from CD4 cell count and baseline viral load, the level of sVL has been repeatedly associated with clinical outcome [13], [14], [48], and is a key marker for later viral control in PHI [49]. The ARSSS predicts these surrogate markers. It could help clinicians identify, at a very early stage, patients with PHI at highest risk of clinical disease progression and opt for an eART. Of note, 70% of the patients in our study had an ARSSS of 1–3, reflecting mild disease; thus, these patients might not need immediate treatment in a resource limited setting, where drug use may have to be triaged.

The potential usefulness of a clinical score to determine the course and prognosis of HIV-1 diseases at the time point of seroconversion was previously demonstrated by other groups [11], [12]: Vanhems at al. reported a significantly increased hazard ratio for CDC category B for PHI patients with at least three symptoms and for category C and death for those with at least five symptoms at the time of acute infection. Lodi et al demonstrated that a clinical severe illness (i.e., bronchitis, pneumonia, oral candidiasis, thrombocytopenia, viral meningitis/encephalitis) at seroconversion was significantly associated with an increased risk of AIDS and death. However, both studies included data from an era when ART was not yet available or eART was not generally recommended. Since withholding cART has been unethical in our study period, we focused on assessing our ARSSS with regards to predict the best studied surrogate markers for HIV-1 disease progression: CD4^+^ cell count and viral load.

One could argue that the composition of our *a priori* defined score is arbitrary and a predictive model is needed. However, there is evidence that useful algorithms can be developed without prior statistical evaluation of individual parameters and formulas. An educated guess based on experience of investigators and knowledge of the literature for selection of variables seem often superior, because they are not affected by accidents of sampling [50], [51]. A classic application of this approach is the *Apgar* test which is still used in clinical practice [52]. The practicability of ARSSS in clinical routine is given by its simple composition and lack of expensive or time-consuming laboratory variables.

An interesting finding which supports the clinical value of the ARSSS in selecting patients for early treatment is that in contrast to the sVL in treatment-naïve patients, no correlation was found with sVL of patients having undergone eART with subsequent treatment interruption. It has been clearly shown in several studies that eART during PHI leads to a transient reduction of the viral setpoint by approximately a factor of 10 when compared to deferred therapy [2], [3] and the VISCONTI study even found some individuals who controlled HIV-RNA spontaneously after interrupting long term eART [53]. The loss of correlation with the ARSSS suggests that eART has a strong and, to a certain extent, sustainable effect and can overrule the negative association of ARSSS with baseline CD4^+^ cell count, baseline VL and sVL that was observed in untreated patients. However, this absence of a signal could also be due to the limited power (only 40 patients with structured treatment interruption).

An additional value of the ARSSS lays in the fact that this score captures patients at highest risk for HIV transmission. Several studies have demonstrated that the risk of HIV transmission increases with higher viral load [54], [55] and the ARSSS itself correlates with this marker. We therefore conclude that initiation of eART in patients with a high ARSSS would also include individuals at higher risk for HIV transmission. We acknowledge that - besides having a high viral load - additional factors determine the infectivity of an individual (e.g., co-infections, sexual high-risk behavior). However, data supports that sexual transmitted infections may increase baseline viral load in HIV infected individuals [56] and therefore are integrated in the ARSSS to a certain extent.

Recently, it has been demonstrated that viral genetic traits can explain up to 50% of viral setpoint variation in HIV-1 infected individuals [57]. On the other hand, host genome-wide association studies showed that variation in the HLA-B and HLA-C genes explain up to 20% of variation in viral control, with little or no impact of human genetic variants located elsewhere in the genome [41]. The vast majority of all these studies, however, were carried out in chronically infected patients. To date, neither viral nor host genetic factors are routinely used in patient management, other than drug resistance testing and HLA testing for abacavir hypersensitivity [58]. In our human genome analysis, no correlation between ARSSS and HLA-B or C variation was found. This is not surprising, given the small sample size and the relatively modest ability of HLA to predict disease progression. It is conceivable that ARSSS integrates virus and host factors alike and thus may be a simple clinical surrogate for the multifactorial nature of HIV-1 disease progression and useful to estimate an individual's vulnerability for severe disease of a patient with a PHI. Thus the ARSSS could serve as a further puzzle piece to guide the clinician in the decision to start eART and to convince patients from the potential benefit of eART.

Our study has strengths and limitations: Strengths: (i) Patients were seen in one center by a stable study-team and laboratory values were measured in a central laboratory; thus, variability of parameters analyzed could be minimized. The study protocol has been unchanged since 2002. (ii) The study population was homogeneous (Table 2). (iii) All but four patients were mono-infected with R5 tropic viruses as previously published [15]. Limitations: (i) We could only evaluate the ARSSS in the context of the two major surrogate markers for HIV disease progression, but could not assess its impact on clinical endpoints. (ii) A potential bias might be that symptomatic patients presented earlier in general, and baseline CD4^+^ cell count and VL might have been determined earlier during PHI when CD4^+^ cell depletion and VL reached high levels. To circumvent this potential bias, we corrected VL and CD4^+^ cell counts according to the EDI and the significant correlation still remained. (iii) Reporting of clinical symptoms is inherently imprecise, but this problem is reduced by combining several predictors in the ARSSS. (iv) We chose an a priori approach to build our score based on variables selected according to knowledge on the literature and our own experience and did not use a statistical model evaluating individual parameters before building the final score. Potentially, important variables could be missed. It cannot be ruled out that the later strategy would also perform well or even better. However, the selection of variables based on a statistical approach is more prone to selection bias inherent to the data, which is not the case in an a priori approach because selection of the parameters is independent on the dataset that is analyzed using the score. Using both approaches and selecting the better performing score would not be appropriate because they could influence each other. In aggregate, the fact that our a priori built score is predictive of key surrogate markers for HIV-1 progression supports the validity of our approach.

In conclusion, the ARSSS predicts the key surrogate markers for HIV-1 disease progression at a very early stage and thus could be used to identify patients with PHI at highest risk of clinical disease progression and may also serve as a research tool. It should be verified in an independent prospective cohort of PHI patients.
